# Numerical study on the characteristics of roadway failure and instability in coal seam with rock parting

**DOI:** 10.1038/s41598-024-51270-w

**Published:** 2024-01-18

**Authors:** Heng Zhang, Yu-Geng Zhang, Guang-Jian Liu, Ya-Wei Zhu, Xian-Jun Ji, Wen-Hao Cao

**Affiliations:** 1 School of Civil Engineering, Nanyang Institute of Technology, Nanyang, 473004 Henan People’s Republic of China; 2 Henan International Joint Laboratory of Dynamics of Impact and Disaster of Engineering Structures, Nanyang Institute of Technology, Nanyang, 473004 Henan People’s Republic of China; 3 College of Earth Sciences, Guilin University of Technology, Guilin, 541006 Guangxi People’s Republic of China; 4 Institute of Rock Mechanics, Ningbo University, Ningbo, 315211 Zhejiang People’s Republic of China; 5 Collaborative Innovation Center for Prevention and Control of Mountain Geological Hazards of Zhejiang Province, Shaoxing University, Shaoxing, 312099 Zhejiang People’s Republic of China

**Keywords:** Energy science and technology, Engineering

## Abstract

In order to explore the mechanism of rockburst in coal seam with rock parting, a combination of on-site and numerical experiment is used to study the failure and instability process, crack propagation mechanism, and influencing factors. The following four points were addressed: (1) the instability is a process that roadway in coal seam with rock parting go through from stable locking in the initial stress unloading stage to slipping unlocking, and then to spatter ejection in slipping dynamic load disturbance stage. (2) The fracture development caused by unloading excavation of coal seam with rock parting will change from shear crack to tensile crack. In this process, coal-rock contact surface slip and coal-rock fracture are coupled with each other. (3) The greater the mining depth is, the greater the lateral pressure coefficient is, and the higher the rockburst risk is. On the contrary, the lower the risk of rockburst. (4) When choosing the support form of roadway in coal seam with rock parting, the two supporting forms of bolting (cable) and supplementary masonry support should be preferred. The results enrich the theory of the dynamics of surrounding rock fracture in coal mine, further clarify the potential dangers to mining-area roadways and working faces, and provide technical information to ensure the safe and efficient mining of bifurcated coal seam.

## Introduction

Rockburst refers to the dynamic phenomenon of sudden and violent damage to coal and rock masses around the roadway or working face^1^. According to its physical characteristics, rockburst can be divided into three types: strain rockburst, roof-caving rockburst and structural instability rockburst^2,3^. Structural instability rockburst usually occurs in the complex geological structure area of coal seam, in which coal seam bifurcation is an important form.

Due to the different sedimentary ages or geological conditions of the sedimentary areas, one or more rock layers are often formed in coal seam^4,5^. Different from the single stable coal seam structure, the existence of rock parting makes the coal seam structure more complex, and the structural form is transformed from a “single coal mass” structure to a “Coal-Rock parting-Coal” combination structure^6,7^. The complex structure of coal and rock parting combinations will cause the change of the structural properties of coal seam^8–10^. On one hand, the existence of rock parting can change the physical and mechanical properties of the original coal seam, including potential indices such as uniaxial compressive strength, elastic energy index and energy index, all of which influence the rockburst potential of the coal mass. On the other hand, the abnormal change in the coal and rock parting structure easily forms a stress concentration area. When mining activities are carried out in the high stress concentration areas, it is very easy to induce a structural instability rockburst.

In recent years, some scholars have conducted considerable research on the failure and instability mechanism of composite coal and rock and have reached some important conclusions^11–14^. However, these studies focus mainly on the uniaxial dynamic and static loading mechanism, without considering the rockburst induced by unloading and the failure and instability of composite structures. Mining is a dynamic process that gradually transitions from triaxial unloading to uniaxial loading, and this transition cannot be ignored. Other scholars have studied the unloading failure and instability process and precursor signal characteristics of coal/rock masses based on the mining unloading mechanism^15–19^, but these studies targeted only a single coal/rock mass structure without considering the slipping induction of the interface in coal and rock composite structures. Therefore, the lack of specific, in-depth and thorough research on the structural failure and instability of the coal seam with rock parting is an important reason for the frequent rockburst disasters in bifurcated seams in recent years.

To address this research gap, this paper takes the “7.29” rockburst accident of the 1305 longwall face as the research case. This paper adopts the method of combining on-site measurements and numerical experiments to study the failure and instability characteristics and influencing factors of roadway in coal seam with rock parting under unloading. The mechanism of coupling instability of slipping and fracture is discussed, and the method of preventing the occurrence of rockburst induced by failure and instability in coal seam with rock parting is put forward. The research results not only enrich the theory of the deformation and failure of the surrounding rock and the dynamic coal and rock disasters affecting roadways and working faces, but also provide technical information for promoting the safe and efficient mining of bifurcated coal seam.

## Engineering background

### Geographical location

**Figure 1:**
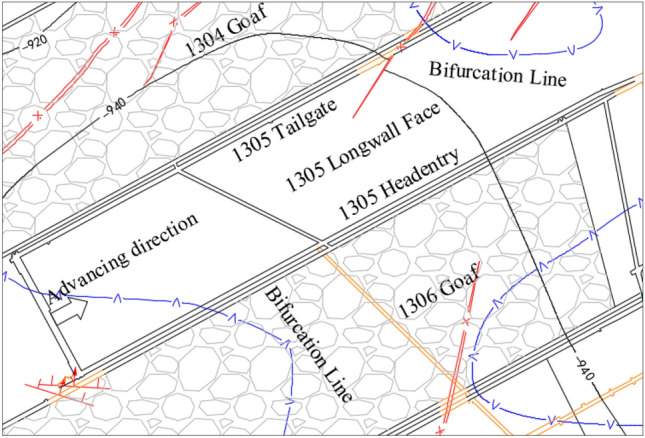
The plane of the 1305 longwall face.

**Figure 2:**
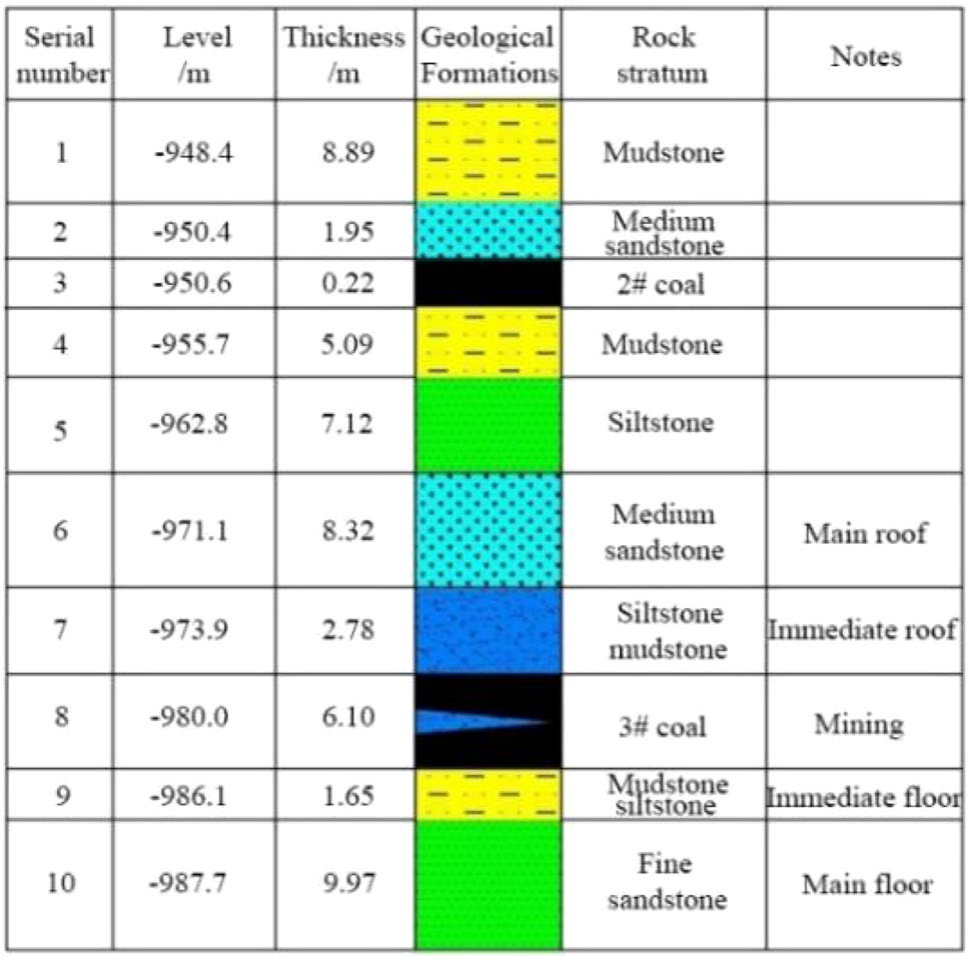
Comprehensive borehole histogram.

The Zhaolou coal mine (ZCM) is located in Heze City, Shandong Province, China. The 1305 longwall face is located in the first mining area of the ZCM, adjacent to the eastern downhill track, extending west to the boundary of the seventh mining area, to the 1304 goaf (Stoppage time: December 25, 2011) to the north, and the 1306 goaf (Stoppage time: January 24, 2013) and 1307 goaf (Stoppage time: July 25, 2015) to the south. The 1305 longwall face is an island working face. The elevation of the 1305 longwall face is − 960 to − 918 m, the average mining depth is 980 m, and the ground elevation is + 43.21 to + 44.34 m. The average strike length of the longwall face is 573 m, the inclined length is 136.7 m, and the coal pillar width is 5 m. The plane of the 1305 longwall face is shown in Fig. 1. Comprehensive borehole histogram is shown in Fig. 2.

### Geological conditions

The mining #3 coal seam of the 1305 longwall face has an inclination angle of 0–11°, with an average of 3°; the thickness of the coal seam is 2.1–8.6 m, with an average of 6.02 m; and the Protodyakonov scale of hardness is f = 4–5. There are two bifurcated areas of coal seam #3 within the longwall face, with a total area of 10142.9 m^2^. The first bifurcation area is located near the design stope line, close to the tailgate side, and the bifurcation area is 3115.3 m^2^. The thickness of upper coal seam #3 in the bifurcation area is 0.6–1.8 m, with an average of 1.1 m. The thickness of lower coal seam #3 is 1.2–2.2 m, with an average of 1.6 m. The bifurcation spacing is 0.7–1.8 m, with an average of 1.4 m. The rock parting in the bifurcation area is mudstone. The second bifurcation area is located near the open-off cut of the working face, close to the side of the headentry, and the bifurcation area is 7027.6 m^2^. The thickness of upper coal seam #3 in the bifurcation area is 0.7–1.2 m, with an average of 0.9 m. The thickness of lower coal seam #3 is 2.7–6.4 m, with an average of 4.8 m. The bifurcation spacing is 0.7–14.6 m, with an average of 6.8 m.

### Accident conditions

**Figure 3:**
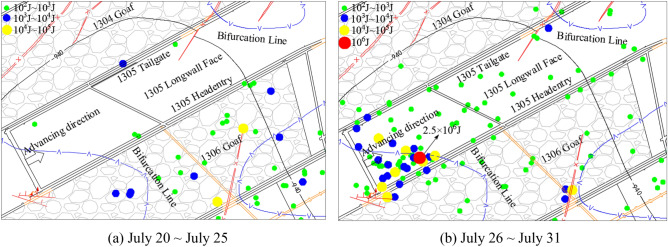
The source location.

On July 29, 2015, rockburst suddenly occurred on the 1305 longwall face. The maximum energy is 2.5 × 10^6^ J. The source location is shown in Fig. 3.

Figure 3a shows the microseismic signal monitoring data from July 20 to July 25. The microseismic signal area monitored before July 25 is mainly inside and near the upper and lower coal seams where the goaf of the 1306 longwall face is located. The number of microseismic signals in front of the working face and the coal wall of the working face and within 150 m of the head entry is very small. At the same time, there are no microseismic signals in and around the upper and lower coal seams at the coal seam bifurcation or in the thickened rock parting near the cutoff. In general, the microseismic signal density and average intensity are very low at this stage.

Figure 3b shows the on-site microseismic monitoring data from July 26 to July 31. The microseismic signal area monitored during this period is located mainly in the coal seam with rock parting surrounded by the bifurcation line of the coal seam. The signal density and average intensity in a large area, including the whole working face and in front of the coal wall of the working face and within 150 m of the headentry, have increased to very high values. Near the rockburst accident, the significant enrichment areas of microseismic signals are in and around the coal seam bifurcation and the upper and lower coal seams near the open-off cut where the rock parting thickens, and the maximum energy is monitored in the rock parting, with a value of 2.5 × 10^6^ J.

### Accident analysis

The 1305 longwall face is an island working face, with a maximum subsidence of 1.7 m on both sides, which is insufficient for mining. The width of the isolated working face is only 136.7 m, and the overall stress of the working face is highly concentrated. Affected by the coal-free area, the open-off cut of the working face is located in the middle of the strike of the goaf on both sides, which is an internal staggered arrangement, and this area is within the stress concentration zone. The average mining depth of the working face is 980 m. There is a coal seam bifurcation zone near the open-off cut. The coal seam bifurcation zone is continuous for more than 300 m. The interior of the bifurcation is a mudstone layer, and there is a stress concentration area near the bifurcation.

Before and after the accident, there was no obvious pressure rise in the roadway on either side of the working face, so it is impossible that the goaf played a major role in the rockburst. The working face advances several meters, and the goaf does not reach the first pressure step of the main roof; therefore, the main roof can’t be the main factor causing this rockburst. Excluding the influence of human factors, according to the previous summary on the geological conditions of the working face, the damage of specific areas after the accident, the monitored microseismic signal distribution and the analysis of the signal energy, It is preliminarily inferred that the rockburst occurred above the coal seam of the working face. The rockburst was caused by the instability of the coal seam under the influence of the rock parting layer. The rockburst mechanism and influencing factors of roadway failure and instability induced by unloading of roadway in coal seam with rock parting are very consistent.

## Numerical model and parameter selection

### Numerical model

**Figure 4:**
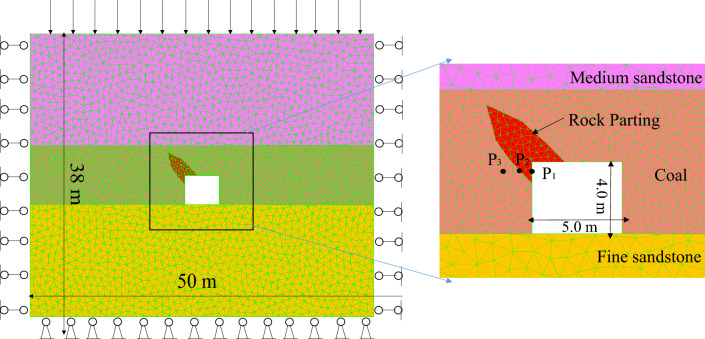
Numerical model of roadway in coal seam with rock parting.

Based on the characteristics of rock partings in the ZCM 1305 longwall face, the UDEC-Trigon method was adopted to establish a numerical model of roadway in coal seam with rock parting, as shown in Fig. 4.

The model size is 50 m × 38 m, and the roadway width is 5.0 m × 4.0 m. The model is divided into three layers using the “Crack” command, where the upper layer is medium-grained sandstone with an average side length of 1.0 m; the middle layer is coal, with an average side length of 0.4 m; and the bottom layer is a fine sandstone floor, with an average side length of 1.0 m. The “Table” command was used to define the wedge-shaped rock parting area and set the rock parting lithology as sandy mudstone. The side length of the rock block is consistent with that of the coal block to eliminate the influence of the change of the block size on the stress transfer.

#### Microparameter selection

The parameter selection of the UDEC numerical model includes blocks and joints. Among these parameters, the linear elastic constitutive equation is selected as the block model. During the simulation, the accumulated elastic energy of the block will be released in the form of kinetic energy, and the block will be thrown out. The linear elastic constitutive equation can ignore the plastic deformation of the material, thereby eliminating the influence of the plastic deformation energy on the accumulation and release of strain energy. The coulomb slip with residual strength is used in the joint model, which can describe the residual strength after the fracture of the contact surface and is more consistent with the actual situation of coal and rock fracture on site. The micromechanical parameters of the blocks and joints selected by the model are shown in Tables 1 and 2^12^.

**Table 1:** Micromechanical parameters of block.

Material	Density kg/m^3^	Bulk modulus GPa	Shear modulus GPa
Medium sandstone	2600	15.0	8.95
Coal	1400	5.0	3.0
Fine sandstone	2600	10.0	5.96
Rock parting	2300	6.3	4.0

**Table 2:** Micromechanical parameters of contact face.

Material	kn/ GPa m	ks/ GPa m	Coh MPa	Fri °	Ten. strength MPa	Res. coh MPa	Res. fri °	Res. ten. strength MPa
Medium sandstone	72	28.8	14	42	11.2	0	32	0
Coal	24.4	9.76	4.0	36	2.5	0	25	0
Fine sandstone	48	19.2	20	42	14.0	0	30	0
Rock parting	28	11.2	3.0	30	2.0	0	25	0
Interface	24.4	9.76	0.5	25	0.5	0	20	0

#### Boundary condition setting

Horizontal displacements are fixed on the left and right boundaries of the model, horizontal and vertical displacements are fixed on the lower boundary, and vertical stress is applied on the upper boundary to simulate the geostatic stress of the overburden. The horizontal stress is obtained from the corresponding multiple of the gravity stress according to experience. To study the failure and instability process of roadway in coal seam with rock parting, the actual crustal stress test results in the no. 1 mining district of the ZCM are used for numerical calculation. The initial crustal stresses in the X-, Y- and Z-directions are set at 32 MPa, 25 MPa and 25 MPa, respectively.

### Numerical experiment

After roadway unloading and excavation, the road will first experience a slow stress release process. In this process, the elastic energy release of near-field coal and rock mass to far-field coal and rock mass is a constrained process. With the unloading time increasing, the constraint load of the near-field roadway decreases gradually until the constraint load is completely unloaded. After the stress is completely relieved, the roadway will produce rockburst instability under the action of the slipping dynamic load on the coal and rock parting interface. Therefore, the unloading-induced failure and instability of the coal and rock parting-combined structure can be divided into two stages: the first stage is the initial stress unloading stage, and the second stage is the slipping dynamic load disturbance stage.

Initial stress unloading stage. The slow stress release process is monitored and controlled by employing the “FISH” program. During the simulation operation, 10% of the initial rock stress is unloaded every 3000 steps until the final initial rock stress around the roadway is unloaded to 0. This stage mainly simulates the process of unloading-induced coal and rock parting interface from stable blocking to slipping unlocking after roadway excavation.

Slipping dynamic load disturbance stage. The dynamic analysis model in the UDEC program is used to simulate the evolution of the rockburst instability of the coal and rock parting composite structure under the slipping dynamic load. During the simulation, the damping coefficient is set to 0 to eliminate the blocking effect of the joint on dynamic load transmission. To reduce the influence of the boundary reflected wave under a dynamic load, the boundary condition of the model is set as viscous to simulate an infinite boundary.

## Roadway failure and instability process

### Initial stress unloading stage

**Figure 5:**
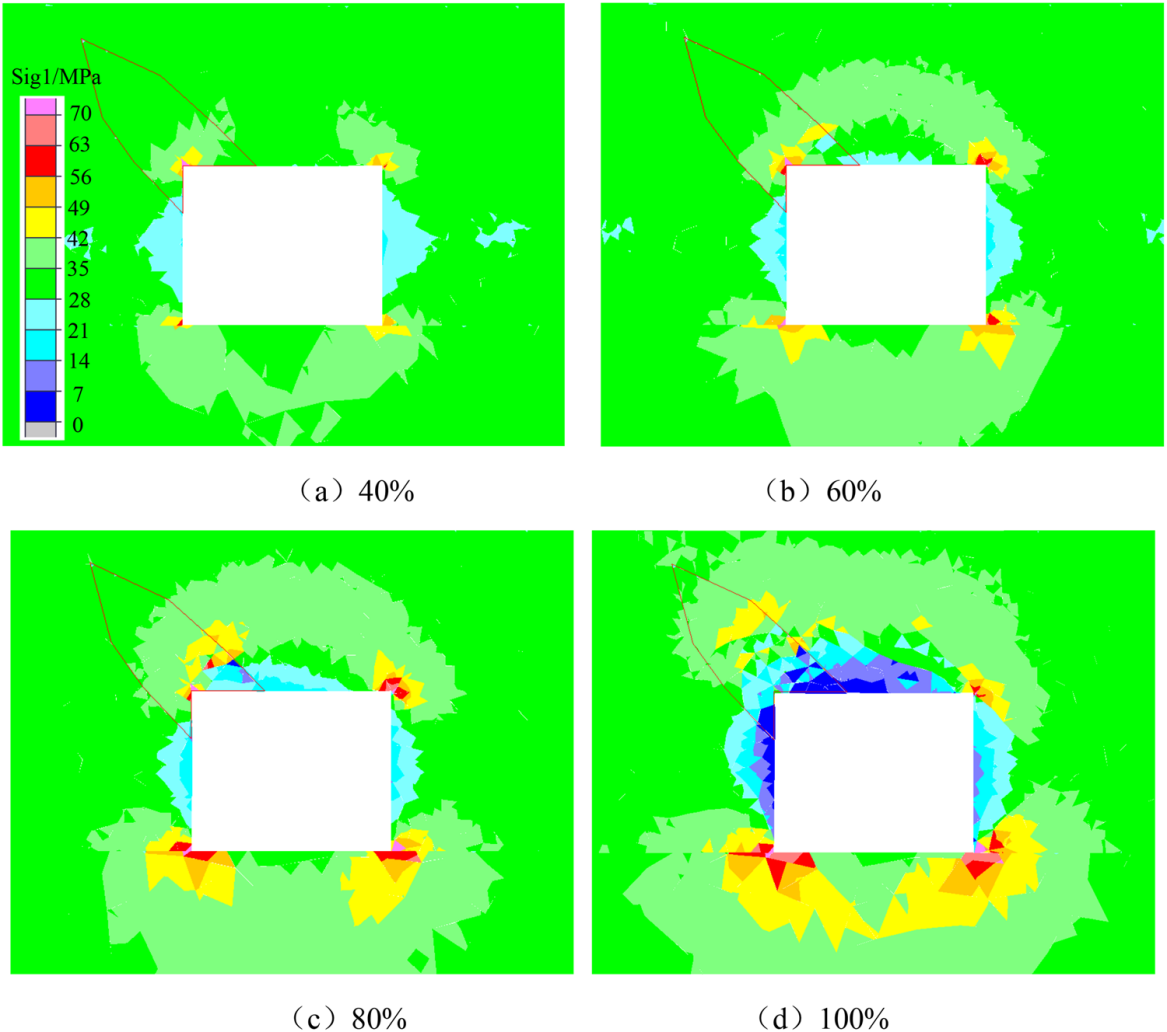
Nephogram of maximum principal stress evolution.

Figure 5 shows the evolution nephogram of the maximum principal stress during the initial stress unloading stage of the roadway in coal seam with rock parting. Figure 5 shows that during the initial unloading, the maximum principal stress around the roadway is obviously concentrated at the four corners of the roadway. When the stress unloading is 40%, the rock parting does not slip, the interface is in a locked state, the maximum principal stress is diagonally distributed, and an obvious “arch” low-stress area occurs at the two sides of the roadway. When the stress is unloaded by 60%, the outer edge of the rock parting is unlocked by slipping, the maximum principal stress is transferred from the corner of the roadway to the rock parting interface, and the range of the low-stress zone at the top and bottom of the roadway and the two sides is further expanded. When the stress is unloaded by 80%, the maximum principal stress on the left side of the roadway is completely transferred to the vicinity of the coal-rock parting interface, and local stress concentration occurs on both sides of the interface. At the same time, a low stress concentration area is generated on the outer edge of the rock parting. When the stress is unloaded at 100%, the force on the outer edge of the roadway is completely relieved, and the maximum principal stress around the rock part is gradually transferred from the outer edge of the rock part to the inner edge. There are obvious low stress concentration areas in the roof, floor and both sides of the roadway. The phenomenon of slip and fracture is obvious, and the stress concentration degree at the corner of the roadway increases considerably, which indicates that unloading can induce the maximum principal stress of the roadway containing rock partings to transfer from the corner of the roadway to the vicinity of the coal and rock parting interface.

**Figure 6:**
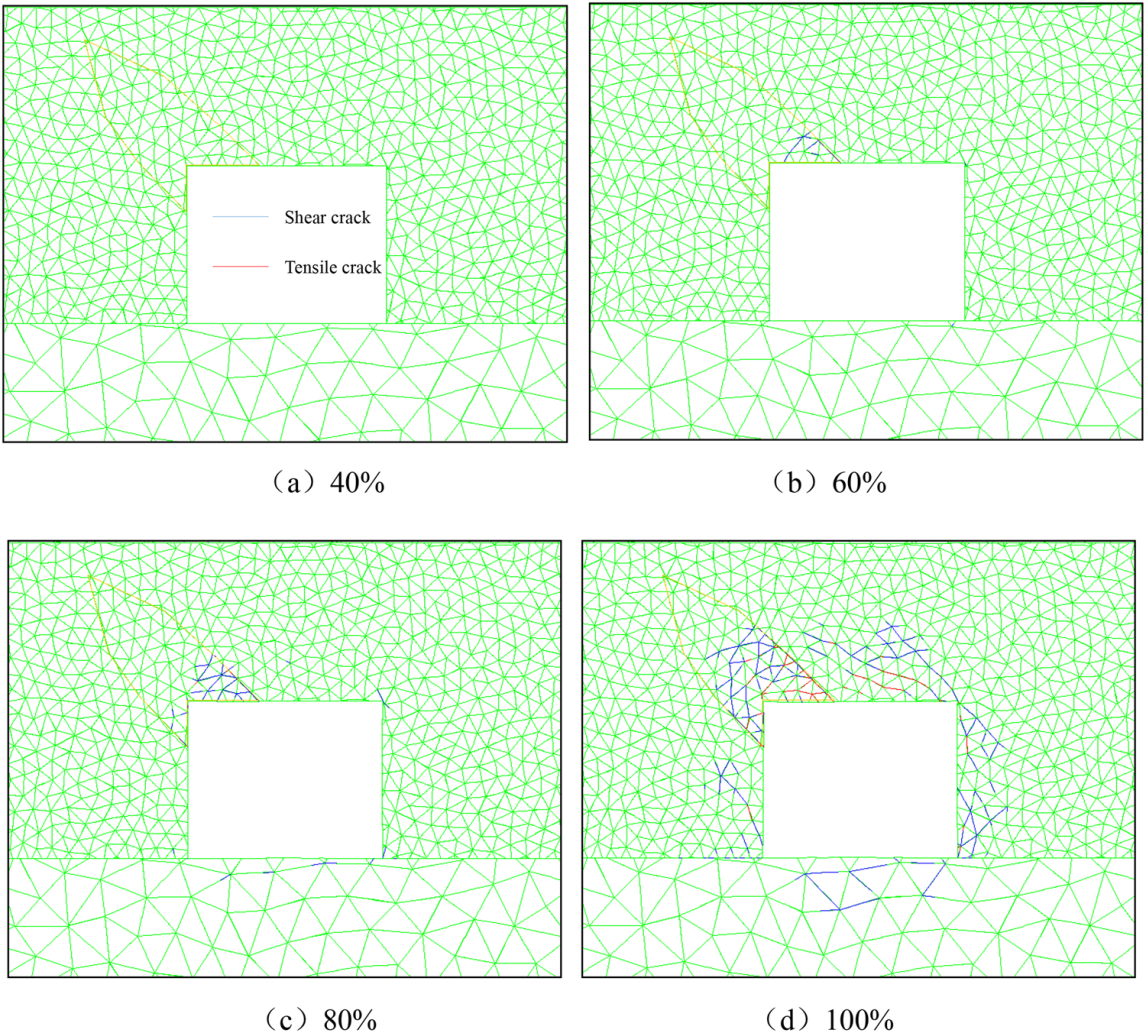
Fracture evolution law of the roadway in coal seam with rock parting.

Figure 6 shows that the stress unloading process of the surrounding rock is accompanied by the generation, expansion and penetration of cracks. The crack first occurs at the interface of coal and rock parting, forming shear cracks that gradually expand and extend to the outer edge of the rock parting. Finally, under the action of tensile cracks, the shear cracks are connected, resulting in the crushing of the rock parting. In the process in which the tensile crack breaks through the outer edge of the rock parting, the shear crack extends to the inside of the rock parting, causing the slip of the internal rock parting block. In Fig. 9, the shear crack concentration is higher in the stress concentration area, and the tensile crack concentration is higher in the stress reduction area, showing that the local stress concentration (shear stress) is the main reason for the slipping instability of the coal and rock parting composite structure along the interface.

**Figure 7:**
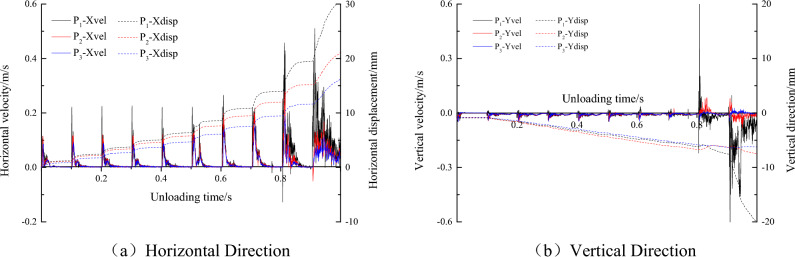
Slip velocity and displacement curves of coal and rock parting.

To explore the influence of unloading on the failure and instability of roadway in coal seam with rock parting, three velocity and displacement monitoring points, P1, P2 and P3, were set on the coal and rock parting interface and on both sides, as shown in Fig. 4. The slip velocity and displacement curves of the coal and rock parting interface and coal and rock mass on both sides at the initial unloading stage are shown in Fig. 7.

Figure 7 shows that the slip velocity and displacement of monitoring point P1 are significantly greater than the slip velocity and displacement of P2 and P3, and as the unloading time increases, the monitoring difference of the three monitoring points gradually increases. When the unloading time is less than 0.8 s, the slip velocity and displacement changes of the three monitoring points are relatively stable, and the coal and rock parting interface near the monitoring point is in a stable locked state. When the unloading time exceeds 0.8 s and the stress unloading degree exceeds 90%, the slip velocity and displacement of monitoring points P2 and P3 are relatively stable, while the monitoring numerical value of monitoring point P1 increases abnormally, which indicates that the rock parting near monitoring point P1 produces obvious slipping and fracture.

### Slipping dynamic load disturbance stage

**Figure 8:**
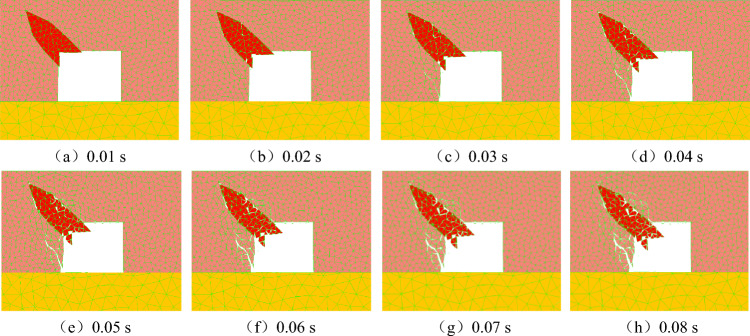
Evolution process of roadway rockburst instability induced by unloading.

During the process of rock parting slippage, a slippage dynamic load will be generated, and the dynamic load can further aggravate the slip and instability of rock parting along the interface and even induce rockburst accidents. Figure 8 shows the evolution process of roadway rockburst instability induced by unloading.

Figure 8 shows that when the dynamic failure time (DFT) is 0.01 s, the rock parting will slip and stagger along the interface, and the rock parting will protrude. When DFT = 0.02 s, the rock parting rock mass begins to produce microfractures, and the outer edge of the rock parting is stripped. When DFT = 0.03 s, the microcracks in the rock parting rock mass begin to extend, converge, connect and penetrate and gradually form macrocracks. At the same time, the rock parting rock mass exhibits obvious failure and obvious unstable slipping. When DFT = 0.04 s, the rock parting fracture body slip into the roadway as a whole, accompanied by an obvious fracture phenomenon. In particular, part of the fracture body separates from the rock parting block and is ejected onto the center of the roadway. Meanwhile, the slipping of the coal and rock parting interface also causes spalling of the coal. When DFT = 0.05 s, along with the further slipping of the coal and rock parting interface, the left side of the roadway fractures, and the spalling range of the coal wall increases further. When DFT = 0.06 s, the slipping and ejection of broken masses are further intensified, and the deformation and failure of the roadway occurs mainly in the area near the rock parting on the left side of the roadway. When DFT = 0.07 s, the microcracks gradually penetrate the roadway roof, and the local fracture coal blocks cave. At the same time, the degree of damage and the scope of the left side of the roadway are further expanded, and the microcracks of the rock parting and inside of the roadway further increase. When DFT = 0.08 s, a large number of fracturing mass fragments are ejected violently, the stripped coal on the left side of the roadway is ejected toward the center of the roadway, and the roof damage is further aggravated. At the same time, microcracks in the combined model expand, converge and coalesce and further cut the block.

**Figure 9:**
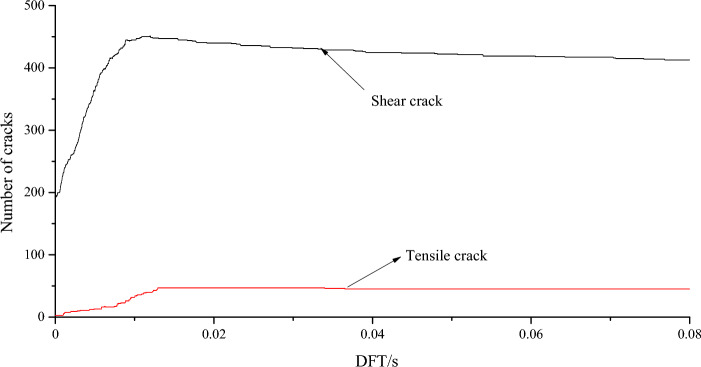
Evolution of shear and tensile cracks in the rock parting area.

Figure 9 shows the evolution of shear and tensile cracks in the rock parting area. When DFT = 0–0.008 s, the number of shear cracks is significantly greater than the number of tensile cracks, and the number of shear cracks increases rapidly, indicating that microcracks in the rock parting develop rapidly under the influence of the slipping dynamic load. When DFT = 0.008–0.013 s, the development rate of shear cracks in the rock parting area gradually slows down and finally reaches the peak value of 450 shear cracks, while the tensile cracks experience rapid growth from 23 to 47 cracks, a twofold increase, indicating that the development of shear cracks occurs earlier than the development of tensile cracks in the process of rock parting slip and instability, and shear cracks can promote the development of tensile cracks to a certain extent. When DFT = 0.013–0.033 s, the shear crack gradually decreases, and the tensile crack remains unchanged at the peak value, indicating that the rock parting blocks begin to separate and are gradually thrown out from the roadway side. It shows that that the joint has been completely destroyed, but the degree of ejection is relatively small, and the rockburst phenomenon is not obvious. When DFT = 0.033–0.08 s, the number of shear and tensile cracks decreases gradually with increasing failure and instability, especially with decreasing shear cracks. It shows that with the increase of dynamic loading time, rock parting is expelled rapidly from coal seam, and the rockburst phenomenon is obviously enhanced.

**Figure 10:**
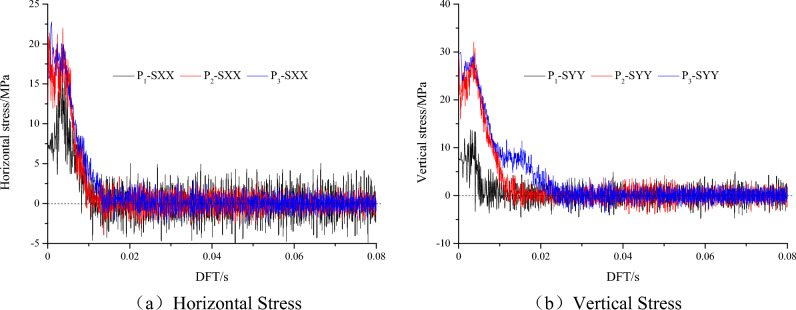
Horizontal and vertical stress evolution curves of monitoring points P1, P2 and P3.

**Figure 11:**
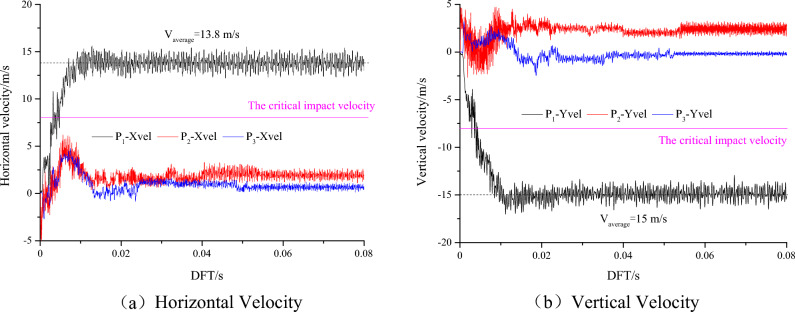
Horizontal and vertical velocity evolution curves of monitoring points P1, P2 and P3.

Figure 10 shows the horizontal and vertical stress evolution curves of monitoring points P1, P2 and P3 under slipping dynamic loading, and Fig. 11 shows the horizontal and vertical vibration velocity evolution curves of monitoring points P1, P2 and P3 under slipping dynamic loading. With increasing dynamic failure time, the stress at the monitoring point gradually decreases, and the vibration speed gradually increases. Finally, the horizontal and vertical stresses of the monitoring point fluctuate at approximately 0 MPa, and the block is thrown out from the roadway. The horizontal vibration velocity of monitoring point P1 gradually increases from 0 to 13.8 m/s, and the vertical vibration velocity gradually increases from 0 to 15 m/s. Finally, the horizontal and vertical velocities fluctuate at approximately 13.8 m/s and 15 m/s, respectively. Both the horizontal and vertical velocities of the block in the fluctuation state are greater than the critical impact velocity (10 m/s). At monitoring point P2, the horizontal and vertical vibration velocities fluctuate violently due to the discontinuous slipping of the coal and rock parting interface, and then the velocity gradually stabilized. The results of monitoring point P3 are similar to the results of monitoring point P2. However, P3 is far from the coal and rock parting interface, the influence of discontinuous slipping of the interface is relatively small, and the horizontal and vertical vibration velocities fluctuate at approximately 0 m/s.

In conclusion, in the initial stress unloading stage, the coal and rock parting mass around the fractures will form a local stress concentration, and the corresponding stress distribution will dynamically evolve. The coal and rock parting interface will experience an instability process from stable locking to slipping unlocking. During the disturbance stage of the slipping load, the coal and rock parting composite structure quickly slips and fractures on the interface under the action of the slipping load, and a large number of fracture coal and rock masses are sprayed outward at high speed, causing rockburst accidents.

## Influence factors of roadway failure and instability

### Influence of mining depth

With increasing coal seam mining depth, the geostatic stress of the overlying rock mass gradually increases, and the mining depth of the coal seam determines the initial stress level of roadway excavation unloading. According to the experimental findings, the higher the initial stress level of unloading is, the more serious the failure and instability of coal and rock will be. Therefore, it is of great value to study the influence of mining depth on the failure and instability of roadway in coal seam with rock parting. To explore how the failure and instability of coal and rock parting composite structures are affected by the depth of the roadway, four numerical models with different depths of 400 m, 600 m, 800 m and 1000 m is set up. The properties of the coal seam, roof and floor strata are assumed to be the same at different mining depths, and the average bulk density of the overlying rocks is 2500 kN/m^3^. The maximum principal stress is the horizontal stress, the lateral pressure coefficient is 1.28, and the lateral pressure coefficient does not change with the depth of the roadway. The direction of the maximum principal stress remains parallel to the direction of the roadway axis. The values of weight stress and structural stress at different mining depths are shown in Table 3.

**Table 3:** Value of gravity stress and tectonic stress under different mining depths.

Mining depth m	Geostatic stress MPa	Tectonic stress MPa	Lateral pressure coefficient
400	10	12.8	1.28
600	15	19.2	1.28
800	20	25.6	1.28
1000	25	32.0	1.28

**Figure 12:**
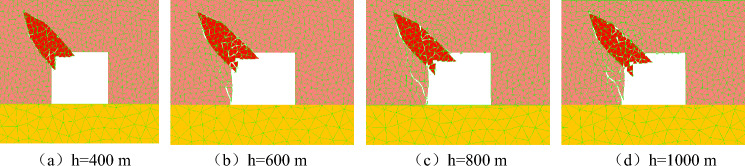
Macrofailure characteristics of roadways at different mining depths.

**Figure 13:**
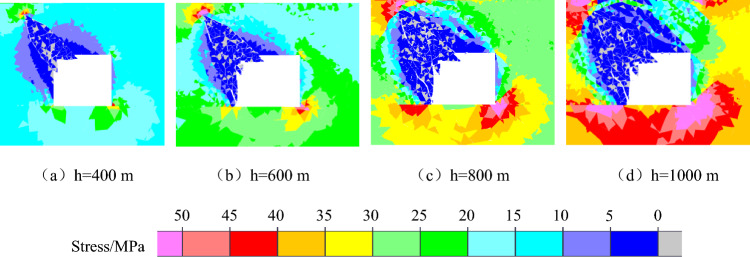
Nephogram of the maximum principal stress distribution under different mining depths.

**Figure 14:**
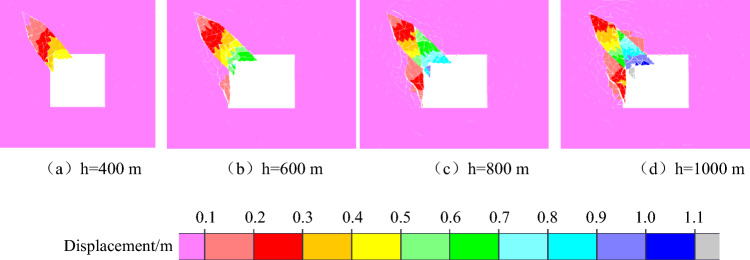
Nephogram of displacement distribution under different mining depths.

**Figure 15:**
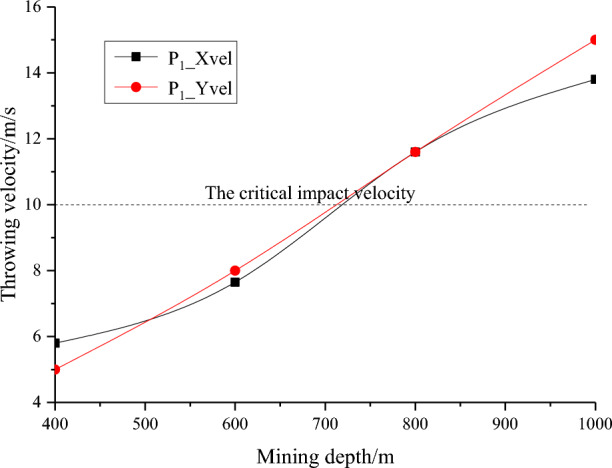
Throwing velocity of rock parting at different mining depths.

The macroscopic failure characteristics, maximum principal stress distribution nephogram, maximum displacement distribution nephogram, and throwing velocity of rock parting blocks at different mining depths during the slipping dynamic load disturbance stage are shown in Figs. 12, 13, 14 and 15.

The following findings can be obtained from Figs. 12, 13, 14 and 15:

With increasing mining depth, the rockburst instability of the roadway in a coal seam with rock parting becomes more obvious. When h = 400 m, rock parting slips occur along the rock parting interface, but the degree of fracture is not high, and no obvious fracture occurs on the roadway wall. When h = 600 m, the rock parting is gradually thrown out, and inclined cracks are formed along the bottom corner of the left side of the roadway, which indicates that the rock parting slip has induced shear failure of the coal body. When h = 800 m or 1000 m, the roadway is affected by the slip of the rock parting, and the left side and roof of the roadway produce obvious coal fracture. Meanwhile, the rock parting is also thrown out, and the rockburst damage of the roadway is obvious. Figure 12 shows that the roadway damage caused by the slipping of the coal and rock parting composite structure is concentrated mainly in the area around the rock parting, and the main form of damage is shear failure.

As the mining depth increases, the far-field stress concentration around the roadway increases significantly, but the stress concentration near the rock parting decreases significantly. Under the influence of rock parting slip and fracture unloading, the maximum principal stress around the roadway is diagonally distributed along the rock parting and the right bottom corner of the roadway. Affected by the mining depth of the coal seam, the maximum principal stress value around the roadway gradually increases, and the range of the stress reduction area around the rock parting gradually expands.

With increasing mining depth, the degree of rock parting fracture gradually increases, and the ejecta displacement of the block gradually increases. When h = 400 m, the maximum displacement of the rock parting ejection is 0.5 m; when h = 1000 m, the maximum displacement reaches 1.1 m, and the crushing degree of the rock parting increases gradually. When h = 400 m, the deep rock parting masses remain structurally stable with the coal body, and the maximum displacement of the rock parting mass is basically 0. When h = 1000 m, the rock parting masses are completely separated from the coal mass because with increasing mining depth, the elastic energy accumulated in the interface between the rock part and coal is relatively high, and the release of elastic energy caused by unloading excavation increases, which will promote the further fracture and slipping of the rock parting.

With increasing mining depth, the throwing velocity of rock parting blocks gradually increases, and the rockburst risk gradually increases. When the mining depth is shallow, the throwing velocity of the rock parting block is low, and the average velocity is less than 10 m/s, which cannot cause rockburst accidents. With increasing mining depth, when h = 700 m, the horizontal and vertical throwing velocities both exceed 10 m/s, which meets the occurrence conditions of rockburst.

In summary, unloading-induced failure and instability of the roadway in a coal seam with rock parting is directly related to the mining depth of the coal seam. The greater the mining depth is, the stronger the rockburst risk of the roadway in coal seam with rock parting. When the mining depth of the coal seam exceeds 700 m, unloading excavation of roadway in coal seam with rock parting can induce rockburst accident.

### Influence of lateral pressure coefficient

The lateral pressure coefficient refers to the ratio of the maximum horizontal stress to the vertical principal stress at a point. In the process of coal seam deposition, the tectonic stress field at the same mining depth is also very different under the influence of horizontal stress extrusion and tension. According to previous studies, the maximum principal stress in the horizontal direction is generally 0.5–5.5 times the vertical stress, and most of the maximum principal stress is concentrated between 0.8 and 1.5.

To explore the influence of the lateral pressure coefficient on the failure and instability of the coal seam with rock parting, the numerical simulation mining depth is 800 m, and four numerical models of lateral pressure coefficient λ = 0.5, 1.0, 1.5 and 2.0 are set. The model assumes that the properties of the coal seam, roof and floor strata are the same, and the average bulk density of the overlying rock layers is 2500 kN/m^3^; that is, the vertical stress is 20 MPa. The horizontal stress changes gradually with the change in the lateral pressure coefficient. The values of dead weight stress and tectonic stress under different lateral pressure coefficients are shown in Table 4.

**Table 4:** Value of gravity stress and tectonic stress under different lateral pressure coefficients.

Lateral pressure coefficient	Self weight stress/MPa	Tectonic stress/MPa	Buried depth/m
0.5	20	10	800
1.0	20	20	800
1.5	20	30	800
2.0	20	40	800

**Figure 16:**
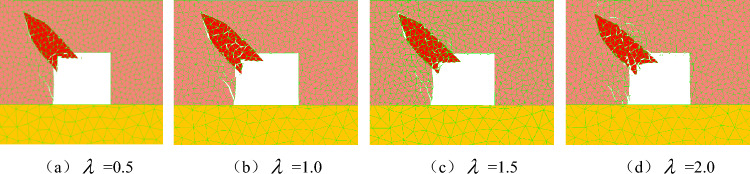
Macrofailure characteristics of roadways under different lateral pressure coefficients.

**Figure 17:**
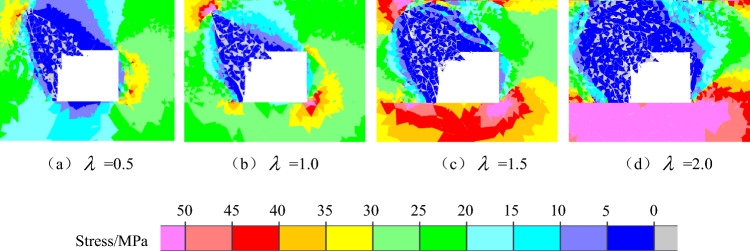
Nephogram of the maximum principal stress distribution under different lateral pressure coefficients.

**Figure 18:**
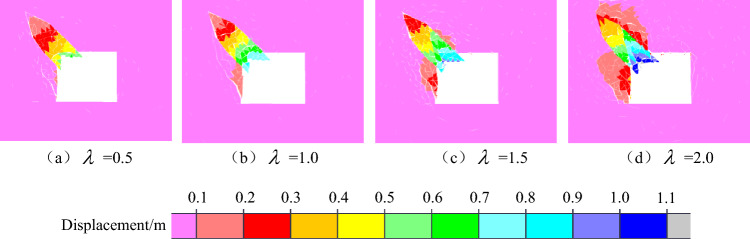
Nephogram of displacement distribution under different lateral pressure coefficients.

**Figure 19:**
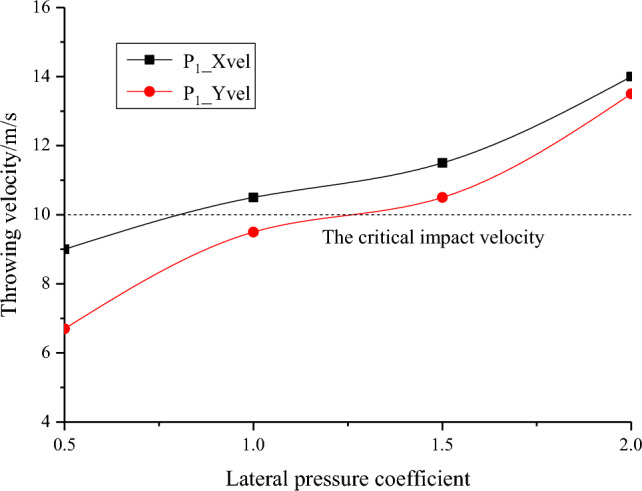
Throwing velocity of rock parting under different lateral pressure coefficients.

The macroscopic failure characteristics, maximum principal stress distribution nephogram, displacement distribution nephogram and throwing velocity of the rock parting block in the slipping dynamic load disturbance stage of the coal seam with rock parting under different lateral pressure coefficients are shown in Figs. 16, 17, 18 and 19.

The following findings can be determined from Figs. 16, 17, 18 and 19.

With the increase in the lateral pressure coefficient, the rockburst instability of the roadway in the coal seam with rock parting becomes more obvious. When λ = 0.5 and 1.0, the maximum principal stress is vertical stress, and the failure and instability characteristics of the roadway are the slipping of the rock parting block and the fracture of coal on the left side of the road, while there is basically no block failure on the right side or roof of the roadway. When λ = 1.5 and 2.0, the maximum principal stress is horizontal stress, and the degree of coal and rock parting fracture on both sides of the roadway increases significantly. In particular, in Fig. 17d, the roof and the right side of the roadway produce obvious block ejections, and the rockburst instability is clear.

With the increase in the lateral pressure coefficient, the far-field stress concentration around the roadway increases, and the near-field stress concentration decreases. When the lateral pressure coefficient is small, the initial stress level of the roadway is low, and the far-field stress concentration is relatively small. For example, when λ = 0.5, the maximum far-field stress is only 40 MPa, and the roadway damage is concentrated mainly in the rock parting area. As the lateral pressure coefficient increases, the damage range of coal and rock parting masses gradually increases. When λ = 1.5, both the right side and the roof of the roadway have a stress reduction area, which is caused by the damage of coal and rock parting inducing the rapid release of the initial elastic energy accumulated in the coal and rock parting, resulting in the reduction of the stress concentration in the failure area. The transfer of near-field stress in the roadway also promotes the rapid concentration of far-field stress. For example, when λ = 2.0, the maximum stress of the coal floor exceeds 50 MPa, and the range of stress concentrations increases significantly.

As the lateral pressure coefficient increases, the degree of rock parting slipping gradually increases, and the coal mass around the roadway is more seriously fragmented. Figure 18 shows that the maximum displacements of rock parting ejection under four different lateral pressure coefficients are 0.6 m, 0.7 m, 0.9 m and 1.1 m. The rock parting ejection displacement gradually increases with increasing lateral pressure coefficient. At the same time, affected by the slipping dynamic load of the rock parting, the fracture degree of coal around the rock parting area also gradually increases with the increase in the lateral pressure coefficient.

With the increase in the lateral pressure coefficient, the throwing velocity of the rock parting block gradually increases. When λ = 0.5, the horizontal velocity of the rock parting block throwing is 9.0 m/s, and the vertical velocity is 6.7 m/s, which does not reach the critical velocity of rockburst instability. When λ = 2.0, the horizontal velocity of the rock parting block throwing is 14 m/s, the vertical velocity is 13.5 m/s, and the roadway produces an obvious rockburst failure. With the increase in the lateral pressure coefficient, the horizontal and vertical velocities of the rock parting block throwing gradually increase.

In summary, the failure and instability of roadway in coal seam with rock parting induced by unloading is also directly related to the lateral pressure coefficient. The greater the lateral pressure coefficient is, the stronger the rockburst risk of the roadway in the coal seam with rock parting. Therefore, in the vicinity of faults, folds and other geological structures, unloading excavation easily induces roadway rockburst accident in coal seam with rock parting. For example, the “11.20” strong mine earthquake in the rock parting occurrence area of the 1307 longwall face of ZCM occurred near the F1715, FX23-2 and FX16 faults(Liu et al., 2019; Lu et al., 2018).

### Influence of support form

**Figure 20:**
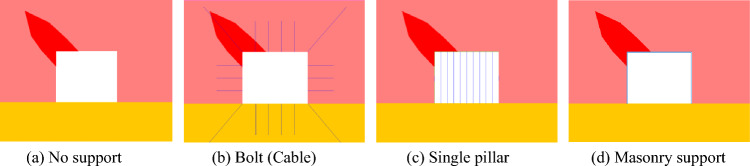
Schematic diagram of different support forms.

Roadway support can greatly improve the stability of the surrounding rock around the roadway, but the stability of the surrounding rock is controlled by different support forms. To explore the effect of support forms on the failure and instability of roadway in coal seam with rock parting, three support forms of no support, bolts (cables), and single pillars and supplementary masonry support were designed for numerical study. The numerical models of the three types of support are shown in Fig. 20. The cable length is 2 m, the rock bolts length is 4 m, and the spacing is 1 m. The rock bolts and cables were represented as a built-in “Cable” element and the masonry supports were represented as a built-in “Beam” element. The parameters of the “Cable” and “Beam” elements are presented in Table 5^20^. The model assumes that the mining depth is 800 m, the average bulk density of the overlying strata is 2500 kN/m^3^, the lateral pressure coefficient is 1.25, and the direction of maximum principal stress is parallel to the axis direction of the roadway.

**Table 5:** Support element properties used in the UDEC Trigon model.

	Property	Value
Cable	Elastic modulus (GPa)	200
	Tensile yield strength (kN)	390
	Stiffness of the grout (N/m/m)	2e9
	Cohesive capacity of the grout (N/m)	4e5
Beam	Elastic modulus (GPa)	200
	Tensile yield strength (MPa)	500
	Compressive yield strength (MPa)	500
	Interface normal stiffness (GPa/m)	10
	Interface shear stiffness (GPa/m)	10

**Figure 21:**
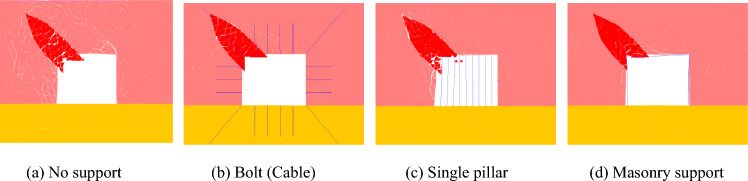
Macrofracture characteristics of roadways under different support forms.

**Figure 22:**
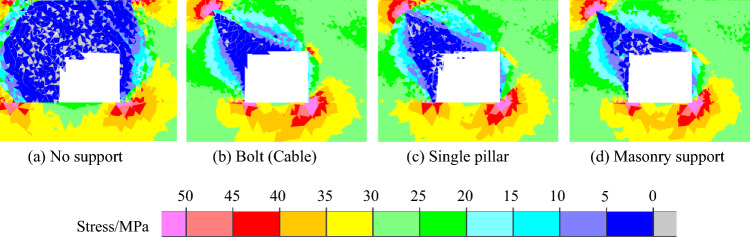
Nephogram of the maximum principal stress distribution under different support forms.

**Figure 23:**
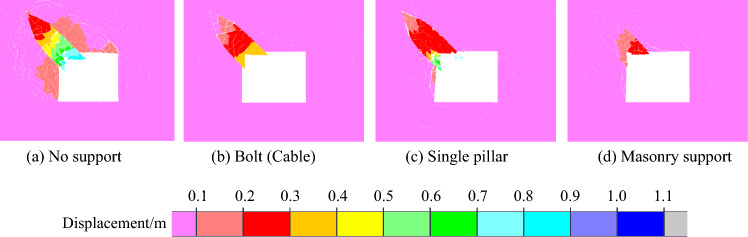
Nephogram of displacement distribution under different support forms.

**Figure 24:**
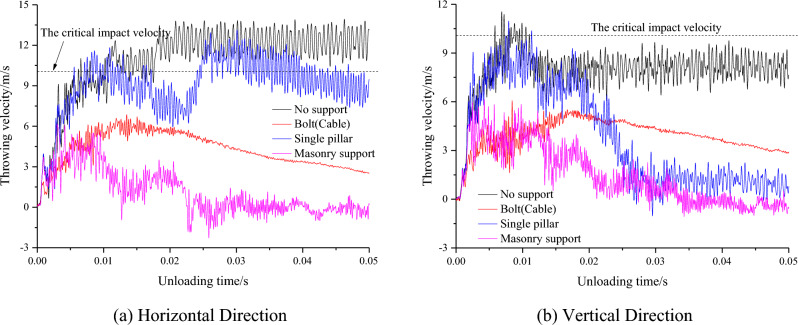
Throwing velocity of rock parting under different support forms.

The macroscopic fracture characteristics, maximum principal stress distribution nephogram, displacement distribution nephogram and rock parting throwing velocity of roadway rockburst instability in coal seam with rock parting under different support forms are shown in Figs. 21, 22, 23 and 24.

The following findings can be determined from Figs. 21, 22, 23 and 24.

When there is no support, there is an obvious block throwing phenomenon in the rock parting and the upper right corner of the roadway, and the roadway rockburst phenomenon is obvious. When the bolt (cable) is supported, the slipping shear stress of the rock parting promotes the bending deformation of the bolt (cable). Under the constraint of bolts (cables), the deformation and failure of roadways are not obvious. With the single pillar supports, the integrity of the roadway roof is better but is affected by the shear stress caused by rock parting slip. The single support on the left side of the roadway exhibits an obvious rightward dumping phenomenon, and at the same time, the left side of the roadway is obviously damaged. When the masonry support is applied, the masonry support can actively bear a certain amount of slip and deformation of the rock parting, and the fracture degree of rock parting is smaller than that of other support forms. However, supplementary masonry is an integral support form. Affected by the deformation of the supplementary masonry, the supporting capacity of the supplementary masonry gradually decreases, and the block failure phenomenon occurs in the roadway roof and the left-side coal mass.

When there is no support, the range of the stress reduction area is large; the coal and rock parting around the roadway is seriously fragmented, especially the position of the roadside and the roof on the side affected by the rock parting; and the coal and rock parting almost completely lose their bearing capacity. With bolt (cable) support, the stress reduction area is concentrated around the rock part and the roof of the roadway. The stress concentration area is located at the far-field boundary of the rock parting and the roadway corner. The deformation and destruction of the roadway are minor, and the stability is considerably enhanced. In the case of a single pillar support, the stress reduction area is concentrated around the rock parting and the left side of the roadway. The stress concentration area is located at the far-field boundary of the rock parting and the corner of the lower right side of the roadway. The analysis was performed because the single pillar cannot be restrained in the horizontal direction, and the roadway side support capacity is insufficient. For the masonry support, the near-field stress of the roadway is considerably smaller than the near-field stress of the bolt (cable) and single pillar support, while the maximum stress in the far-field stress reduction area of the roadway is significantly higher, indicating that the near-field damage of roadways is more serious than the near-field damage of other support forms in the case of masonry support, while the stability of far-field roadways is better.

When the roadway is in the state of no support, the horizontal velocity of lost rock parting reaches 12 m/s, which exceeds the rockburst critical velocity, indicating that the roadway has the risk of rockburst instability and no support. When bolts (cables) are employed, a single pillar and masonry support are used, and the throwing velocity of the rock parting block is obviously reduced. Among these supports, masonry support has the most obvious weakening effect on the throwing velocity of the parting rock, and the weakening effect of a single pillar is the poorest, showing that the three support forms can reduce the risk of rockburst instability of roadway in coal seam with rock partings, and the effect of masonry support is the best, followed by anchor bolts (cables), and the effect of a single pillar is the poorest.

In summary, reasonable support forms play an important role in preventing unloading-induced rockburst disaster accidents in coal seam with rock parting. When selecting the support form of the roadway in coal seam with rock parting, the anchor bolt (cable) and the masonry support form should be preferred.

### Comparison of rockburst between field and numerical simulation

**Figure 25:**
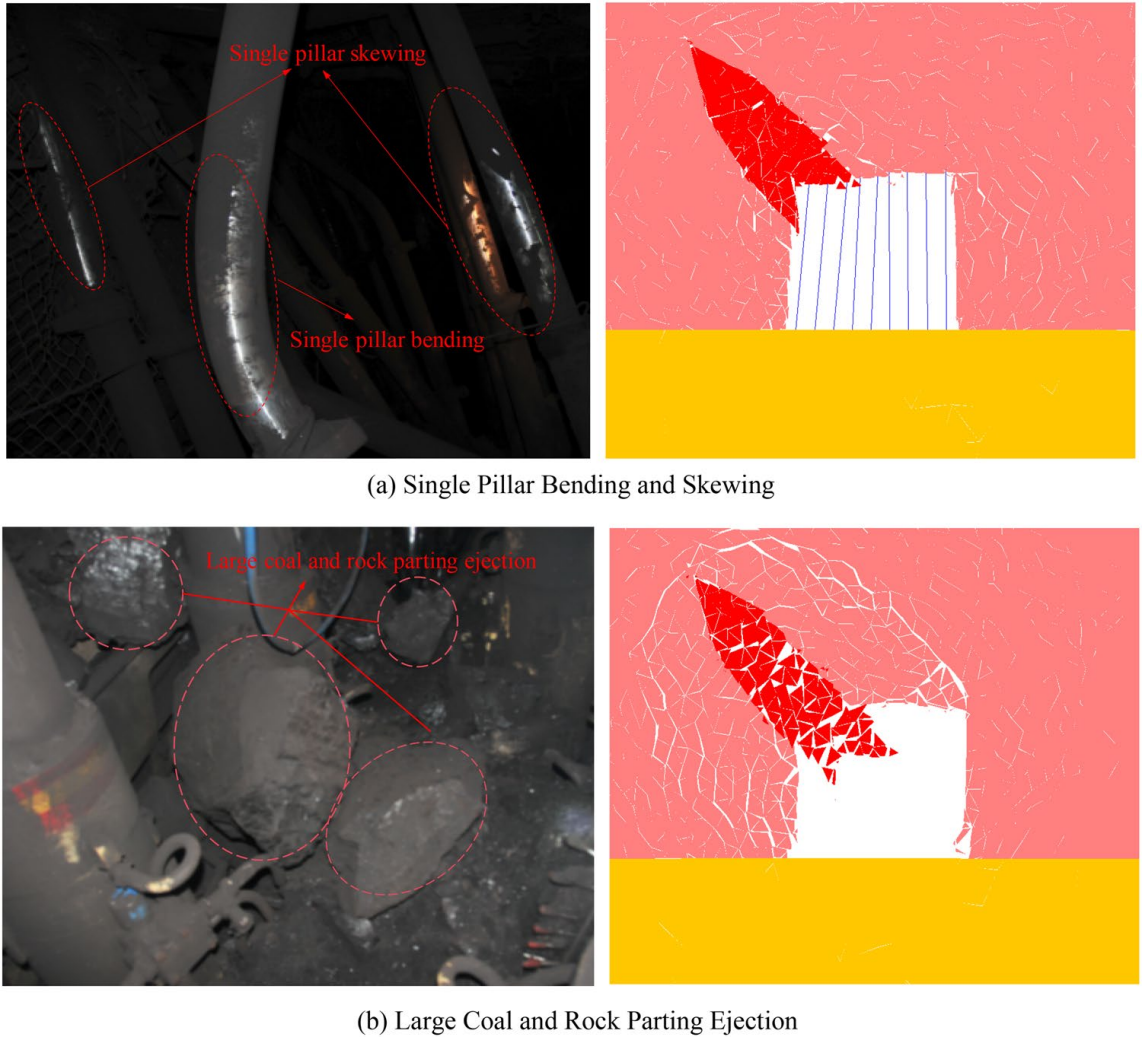
Comparison of field rockburst and numerical results.

At 2:49 on July 29, 2015, a rockburst accident occurred at the 1305 working face of the ZCM, which was related to the presence of the rock parting and was ultimately identified as the slip and instability of the rock parting under the action of high static load stress in the island coal pillar working face. Site photos of the two accidents were selected for comparative analysis with numerical simulation results, as shown in Fig. 25.

Figure 25a shows the bending and skewing of the single pillar. The tailgate of the 1305 longwall face is within 15 m outward along the coal wall, and the roadway height is approximately 4.0 m. The deformation of the two sides is not obvious, the roof has net pockets, and some leaks and some single pillars are skewed. In the range of 15–60 m, the two sides have a large displacement, the maximum displacement is approximately 3.0 m, and the working face has a large lateral displacement. The single pillar part of the advanced support is bent and skewed, and the bottom heave is 0.5–1 m.

Figure 25b shows the throwing out of large coal and rock parts. The 1305 longwall face is bounded by support No. 65 and rockburst upward and downward. There is a phenomenon of large coal and rock parting rushing out between supports No. 60–65. In the process of the numerical test, the throwing out of the rock parting also occurs, which is basically consistent with the actual situation on site.

Through comparative analysis, the numerical simulation results can be seen to be well coupled with the actual failure characteristics, indicating that unloading can induce rockburst accident in coal seam with rock parting. At the same time, the accuracy of the numerical results is also verified.

## Conclusion

1. The failure and instability process of roadway in coal seam with rock parting is divided into two stages: the initial stress unloading stage and the slipping dynamic load disturbance stage. In the initial stress unloading stage, the coal and rock masses around the fissure will form local stress concentrations, and the corresponding stress distribution will evolve dynamically. The coal and rock parting interface experiences an instability process from stable blocking to slipping unlocking. At the disturbance stage of the slipping dynamic load, rapid slipping and fracture occur at the interface of the roadway in coal seam with rock parting, and a large number of pieces of broken coal and rock masses are ejected outward at high speed.
2. The fracture development is gradually transformed from shear cracks to tensile cracks. In this process, slipping and fracture are coupled with each other. In the unloading process, the crack expands rapidly until the rock parting is ejected. This fracture causes slipping dislocation of the internal rock parting along the interface, and the microcracks in the rock mass begin to extend, converge, connect and penetrate. This process further leads to the coal and rock parting interface to slip because of coal wall spalling. As a result, the phenomenon of slipping and ejection of the fracture mass is further intensified.
3. The greater the mining depth is, the greater the lateral pressure coefficient is, and the higher the rockburst risk of the roadway is. On the contrary, the lower the risk of rockburst. Reasonable support systems can improve the safety and reduce the rockburst risk of roadway in coal seam with rock parting.
4. When selecting the support form of the roadway in coal seam with rock parting, bolt (cable) and supplementary masonry support should be preferred.

## Data Availability

The datasets used and analysed during the current study available from the corresponding author on reasonable request.
